# Characterizing Brain Tumor Regions Using Texture Analysis in Magnetic Resonance Imaging

**DOI:** 10.3389/fnins.2021.634926

**Published:** 2021-06-03

**Authors:** Yun Yu, Xi Wu, Jiu Chen, Gong Cheng, Xin Zhang, Cheng Wan, Jie Hu, Shumei Miao, Yuechuchu Yin, Zhongmin Wang, Tao Shan, Shenqi Jing, Wenming Wang, Jianjun Guo, Xinhua Hu, Yun Liu

**Affiliations:** ^1^School of Biomedical Engineering and Informatics, Nanjing Medical University, Nanjing, China; ^2^Institute of Brain Functional Imaging, Nanjing Medical University, Nanjing, China; ^3^National Key Laboratory for Novel Software Technology, Nanjing University, Nanjing, China; ^4^Institute of Medical Informatics and Management, Nanjing Medical University, Nanjing, China; ^5^Department of Neurosurgery, The Affiliated Brain Hospital of Nanjing Medical University, Nanjing, China

**Keywords:** texture analysis, MRI, SVM, brain tumor, t-SNE

## Abstract

**Purpose:**

To extract texture features from magnetic resonance imaging (MRI) scans of patients with brain tumors and use them to train a classification model for supporting an early diagnosis.

**Methods:**

Two groups of regions (control and tumor) were selected from MRI scans of 40 patients with meningioma or glioma. These regions were analyzed to obtain texture features. Statistical analysis was conducted using SPSS (version 20.0), including the Shapiro–Wilk test and Wilcoxon signed-rank test, which were used to test significant differences in each feature between the tumor and healthy regions. T-distributed stochastic neighbor embedding (t-SNE) was used to visualize the data distribution so as to avoid tumor selection bias. The Gini impurity index in random forests (RFs) was used to select the top five out of all features. Based on the five features, three classification models were built respectively with three machine learning classifiers: RF, support vector machine (SVM), and back propagation (BP) neural network.

**Results:**

Sixteen of the 25 features were significantly different between the tumor and healthy areas. Through the Gini impurity index in RFs, standard deviation, first-order moment, variance, third-order absolute moment, and third-order central moment were selected to build the classification model. The classification model trained using the SVM classifier achieved the best performance, with sensitivity, specificity, and area under the curve of 94.04%, 92.3%, and 0.932, respectively.

**Conclusion:**

Texture analysis with an SVM classifier can help differentiate between brain tumor and healthy areas with high speed and accuracy, which would facilitate its clinical application.

## Introduction

Brain cancer remains a diagnostic challenge for clinicians and radiologists because malignant brain tumor cells can invade into the neighboring cells in the brain and spinal cord with fuzzy borders and have a high progression rate (Wild, 2014; Vargo, 2017; Tandel et al., 2019). Treatment of advanced brain tumors is difficult; therefore, early diagnosis is of great importance in clinical settings. The approaches currently employed for the diagnosis of brain tumors include both invasive and noninvasive methods. Although the invasive diagnostic method—biopsy—is viewed as the golden standard for the diagnosis of brain tumors, noninvasive diagnostic methods including magnetic resonance imaging (MRI) are safer and more widely used (Zhao and Jia, 2016). Determination of the accurate location and segmentation of the brain tumor on MRI scans are essential for treatment planning (Mahaley et al., 1989). Several studies have found MRI features capable of differentiating between the tumor and healthy regions (May et al., 1991; Drape et al., 1992; Mullen and Huang, 2017). However, in most cases, the diagnostic accuracy only depends on the proficiency of the medical practitioner reading the MRI scan (Hayward et al., 2008). Many complex patterns, also called image textures, remain imperceptible to the naked eye. Texture analysis is a practical approach for image pattern recognition by extracting objective information through the analysis of the spatial distribution of intensity variations on images (Haralick and Shanmugam, 1973; Haralick, 1979). Furthermore, several studies have confirmed the efficiency of texture analysis (Bayanati et al., 2015; Hodgdon et al., 2015; Skogen et al., 2016).

To increase the diagnostic precision and efficiency, many computer-assisted methods have been developed and introduced, including machine learning (ML) and deep learning (DL) (Zhao and Jia, 2016; Boissoneault et al., 2017; Salvador et al., 2017). Texture analysis combined with ML methods has been widely used to evaluate medical images and yielded promising results (Fetit et al., 2015; Li et al., 2016; Bisdas et al., 2018). However, to the best of our knowledge, there are a few reports on the use of t-distributed stochastic neighbor embedding (t-SNE), which is a new dimensionality reduction and visualization technique to foresee data for preventing problems such as incorrect marking of images and that can help increase the accuracy of the classification.

We hypothesized that some texture features acquired from MRI scans would serve as classification features and markedly improve classification efficiency. To test our hypothesis, the Gini impurity index in the random forests (RFs) was applied to select features, which were then used to develop classification models. Finally, the performance of the features and the models in confirming our hypothesis was assessed.

## Subjects and Methods

### Subjects

The data used were collected from the Affiliated Nanjing Brain Hospital of Nanjing Medical University. Patients in whom meningioma or glioma was histopathologically confirmed between January 2014 and December 2014 were selected. In all, 40 patients (average age: 51.10 years) comprising 22 men (average age: 52.36 years) and 18 women (average age: 47.33 years) were included. The exclusion criteria were as follows: (1) presence of other organic mental disorders and nervous system diseases and (2) a history of major physical illnesses. All of the patients met the above criteria. The study was approved by the medical ethics committee of Nanjing Medical University. All patients provided signed written informed consent.

### MRI Acquisition

All images were acquired using a 3T Siemens MRI system. The patients were instructed to relax, keep their eyes closed, stay awake, and remain still. Patient compliance was confirmed after scanning was completed. The images were recorded axially for 6 min by using an echo-planar imaging sequence with the following parameters: TR = 1900 ms, TE = 2.49 ms, slice thickness = 1 mm, flip angle = 90°, and matrix size = 256 × 256. All patients underwent MRI without reporting discomfort during or after the procedure.

### Classification Based on 25 Texture Features

#### Preparation Before Classification

For the experimental preparation, the raw sample image format was changed from DICOM to JPG. In the texture analysis, the tumor region in the coronal MRI image was selected as the experimental group, and the symmetrical healthy region on the other side of the brain was selected as the control group. There were 40 tumor regions in the experimental group and 40 healthy regions in the control group. In each group, 25 texture features (belonging to three categories) were calculated, as shown in Table 1.

**TABLE 1:** The selected texture features in the three categories.

Category (-based parameters)	Texture features
Histogram	(1) First-order moment; (2) second-order moment; (3) third-order moment; (4) fourth-order moment; (5) the central moment of the four features; (6) the absolute moment of the four features
Run-length matrix	(1) Long run emphasis; (2) short run emphasis; (3) gray level nonuniformity; (4) total run-length percentage
Co-occurrence matrix	(1) Energy; (2) inertia moment; (3) correlation; (4) entropy; (5) mean; (6) variance; (7) standard deviation; (8) homogeneity; (9) dissimilarity

The 25 texture features were recorded as mean ± SD. Statistical analysis was performed using SPSS (version 20.0), including the Shapiro–Wilk test and Wilcoxon signed-rank test, which was used for testing significant differences in each feature between tumors and healthy areas. Meanwhile, an RF model was employed to predict whether each sample was a tumor or a healthy area and for importance rankings of 25 texture features according to the Gini impurity index in the RF (Menze et al., 2009; Liu et al., 2018). All texture features were selected as predictors to compare the experimental results from the Wilcoxon signed-rank test and RF prediction. In addition, t-SNE, a new dimension reduction and visualization technique for high-dimensionality data, was performed in the exploratory analysis (Li et al., 2017). It was applied to all 40 pairs of samples with 25 features to delete the samples that would apparently have a negative effect on the latter classification.

#### Classification

The samples were randomly divided into training (70%) and test sets (30%). This was iterated five times to provide five unique training and testing groups. The training set was used to generate classification models with three different classifiers: RF, BP, and SVM.

The RF is fast, is flexible, and has become a standard tool in biomedical informatics. Each classifier in the ensemble is a decision tree classifier and is generated using random selection of attributes at each node to determine the split. During classification, each tree votes, and the most popular class is returned.

The BP iteratively processes a set of training tuples and compares the network’s prediction with the actual known target value. For each training tuple, the weights are modified to minimize the mean squared error between the network’s prediction and the actual target value. Modifications are made in the backwards direction. The process will reach the terminating condition when the error is very small.

The SVM is a classification method for both linear and nonlinear data. It uses nonlinear mapping to transform the original training data into a higher dimension. With the new dimension, it searches for the linear optimal separating hyperplane. With an appropriate nonlinear mapping to a sufficiently high dimension, data from two classes can always be separated by a hyperplane. SVM finds this hyperplane using support vectors and margins.

Four indexes were used to evaluate each model, including the area under the curve (AUC), error rate, sensitivity, and specificity. Moreover, the receiver operating characteristic (ROC) curve was constructed for each model.

## Results

### Texture Feature Analysis

Using the Wilcoxon signed-rank test, 25 texture features could reveal regions with higher and lower texture values when comparing the experimental (tumor region) and control groups (health region), as shown in Tables 2–4. We obtained the importance rankings of the 25 texture features according to the Gini impurity index in the RF with a training set (80%). The top five features were standard deviation, first-order moment, variance, third-order absolute moment, and third-order central moment, as shown in Table 5.

**TABLE 2:** Wilcoxon signed-rank test results (histogram).

No.	Texture features	Tumor region	Health region	*P*-value
1	First-order moment	10.661 ± 3.414	3.664 ± 5.111	0.000*
2	First-order central moment	2.327 ± 0.280	1.071 ± 0.934	0.000*
3	First-order absolute moment	2.327 ± 1.280	1. 071 ± 0.934	0.000*
4	Second-order moment	159.912 ± 51.205	54.958 ± 76.657	0.000*
5	Second-order central moment	19,442.302 ± 13,715.926	6,490.148 ± 13,691.211	0.000*
6	Second-order absolute moment	19,442.302 ± 13,715.926	6,490.148 ± 13,691.211	0.000*
7	Third-order moment	2,398.676 ± 768.069	824.368 ± 1,149.855	0.000*
8	Third-order central moment	−14,586,918,376.546 ± 12,193,828,145.790	−5,165,830,452.742 ± 11,313,615,571.915	0.000*
9	Third-order absolute moment	14,586,918,376.546 ± 12,193,828,145.790	5,165,830,452.768 ± 11,313,615,571.903	0.000*
10	Fourth-order moment	35,980.138 ± 11,521.031	12,365.514 ± 17,247.826	0.000*
11	Fourth-order central moment	2,201,613,996,782,910,720.000 ± 2,158,254,619,227,325,440.000	827,213,133,922,581,760.000 ± 1,882,686,712,587,172,610.000	0.000*
12	Fourth-order absolute moment	2,201,613,996,782,910,720.000 ± 2,158,254,619,227,325,440.000	827,213,133,922,581,760.000 ± 1,882,686,712,587,172,610.000	0.000*

*means that this data shows significance.

**TABLE 3:** Wilcoxon signed-rank test results (run-length matrix).

No.	Texture features	Tumor region	Healthy region	*P*-value
1	Long run emphasis	628.833 ± 512.533	782.519 ± 639.539	0.188
2	Short run emphasis	0.228 ± 0.076	0.234 ± 0.100	0.582
3	Total run-length percentage	0.084 ± 0.0385	0.078 ± 0.046	0.476
4	Gray level nonuniformity	207.211 ± 129.511	154.085 ± 86.213	0.011*

*means that this data shows significance.

**TABLE 4:** Wilcoxon signed-rank test results (co-occurrence matrix).

No.	Texture features	Tumor region	Healthy region	*P*-value
1	Energy	0.637 ± 0.198	0.812 ± 0.161	0.000*
2	Entropy	0.663 ± 0.328	0.404 ± 0.288	0.002*
3	Inertia moment	15.809 ± 10.322	13.5043 ± 12.0488	0.313
4	Correlation	0.105 ± 0.276	0.093 ± 0.150	0.026*
5	Homogeneity	0.939 ± 0.040	0.948 ± 0.047	0.313
6	Dissimilarity	0.988 ± 0.645	0.844 ± 0.753	0.313
7	Mean	12.444 ± 3.645	4.876 ± 5.465	0.000*
8	Variance	152.277 ± 47.371	53.902 ± 71.028	0.000*
9	Standard deviation	15.004 ± 2.084	8.575 ± 4.677	0.000*

*means that this data shows significance.

**TABLE 5:** Gini impurity index in the RF.

No.	Texture features	Mean decrease Gini	Rank
1	First-order moment	1.7897453	7
2	First-order central moment	0.8495841	16
3	First-order absolute moment	0.7873654	19
4	Second-order moment	1.8026458	6
5	Second-order central moment	1.5147000	10
6	Second-order absolute moment	1.3757714	13
7	Third-order moment	1.6430335	9
8	Third-order central moment	1.8037972	5
9	Third-order absolute moment	1.8316361	4
10	Fourth-order moment	2.0892720	2
11	Fourth-order central moment	1.0609800	14
12	Fourth-order absolute moment	1.4124922	12
13	Energy	0.4763909	25
14	Entropy	0.5165798	23
15	Inertia moment	0.7618972	20
16	Correlation	0.7355067	21
17	Homogeneity	0.8385106	17
18	Dissimilarity	0.7343357	22
19	Mean	1.7526929	8
20	Variance	1.9482926	3
21	Standard deviation	3.1126458	1
22	Long-run emphasis	0.8664384	15
23	Short-run emphasis	1.4297721	11
24	Total run-length percentage	0.8314745	18
25	Gray-level nonuniformity	0.4918337	24

**FIGURE 1:**
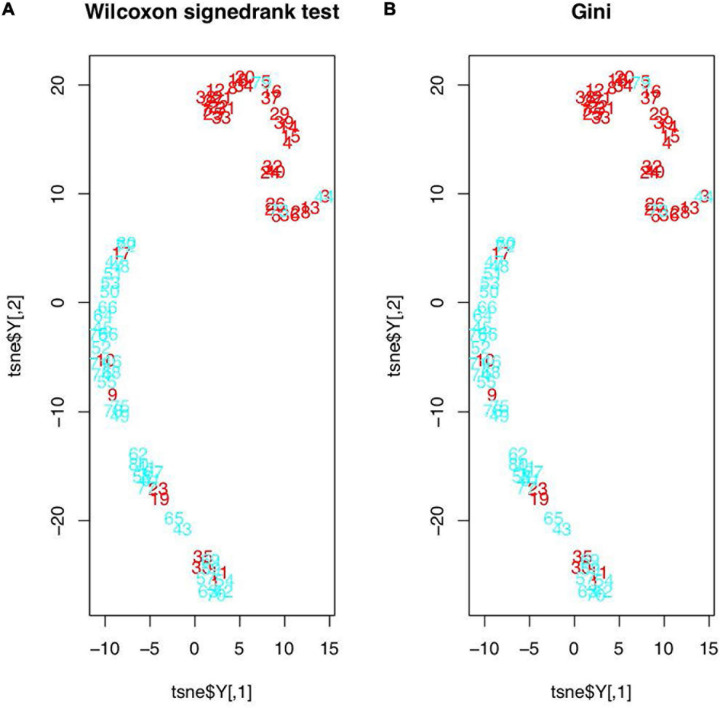
Feature distributions after t-SNE: **(A)** features based on the Wilcoxon signed-rank test; **(B)** features based on the RF’s importance rankings (red figures represent the healthy regions and blue figures represent the tumor regions).

The t-SNE test results are shown in Figure 1. In Figure 1A, the original features were those found in the Wilcoxon signed-rank test (19 features in total), and in Figure 1B, the original features were the top five features determined in the RF’s importance rankings. However, the data distributions after t-SNE were similar. All samples were evidently divided into two clusters, except 12 samples (1, 9, 10, 11, 17, 19, 23, 30, 35, 44, 73, and 79), which were seemingly distributed mistakenly. In addition, *t*-test was used to examine 40 samples to determine whether their features were relatively different between the tumor and healthy regions. We found that the mean *P*-value was 0.2390645. The *P*-values of seven samples—1, 9, 10, 19, 23, 30, and 35—were greater than the mean *P*-value, and these samples were also mistakenly distributed in the t-SNE picture and were deleted.

### Classifier Evaluation

On the basis of the results obtained above, we selected the five features (standard deviation, first-order moment, variance, third-order absolute moment, and third-order central moment) identified in the RF to set up classifiers, which helped save calculation time and resources. Three classification models (RF, SVM, and BP) were applied, and five features were used to train each classifier. A detailed summary of the model’s performance is presented in Table 6.

**TABLE 6:** Three classifiers evaluation.

Classifiers	AUC	Error rate (%)	Sensitivity (%)	Specificity (%)
RF	0.856	14.1	82.8	88.3
SVM	0.932	6.9	94.04	92.3
BP	0.884	11.4	87.2	89.6

**FIGURE 2:**
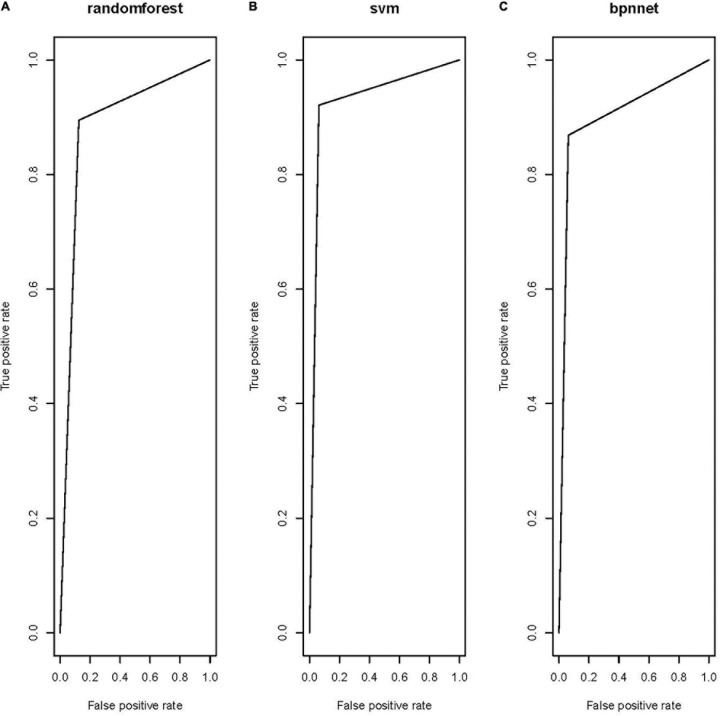
Receiver operating characteristic of the three models: **(A)** RF model, **(B)** SVM model, and **(C)** BP model.

All three models showed satisfactory AUCs of 0.85–0.95. The RF and the BP shared a similar performance based on the AUC, error rate, sensitivity, and specificity. The model trained by the SVM classifier demonstrated the best performance among the three models, with markedly better AUC, error rate, sensitivity, and specificity, indicating that this model could correctly classify the tumor and healthy regions. Receiver operating characteristic (ROC) curves were constructed for the three models to compare their performance directly, as shown in Figure 2.

## Discussion

Some studies have reported the same methods to select features, and the validity of this approach has been proven. Wang et al. (2018) evaluated the importance of spectra lines based on RFs and then used a support vector machine (SVM) classifier to classify the laser-induced plasma spectra (LIBS) of bacteria species. The primary objective of this study was to characterize tumor regions using MRI-based texture analysis. We used texture analysis to compute 25 texture features from MRI images. Using the Wilcoxon signed-rank test, we confirmed that 19 texture features of the total 25 features were different between the healthy and tumor regions. Using the t-SNE technique, the dataset was divided into two clusters, indicating that there is a high possibility to set up a classification model with these 19 features. However, training a model with high-dimensionality data requires a lot of time and space. To facilitate faster and more accurate classification, the importance rankings of the features in the RF were calculated, and the top five features were found to show the same classification effectiveness as the 19 features selected before.

The images for the t-SNE test results showed some seemingly noisy dots. Considering the possibility that all mistakenly distributed samples may be deleted incorrectly, the *t*-test was applied to generally examine whether the healthy and tumor regions showed significant differences in the 25 texture features for each sample. To determine the modified number of samples that would be deleted, the mean *P*-value was set as the deletion standard, and seven samples were excluded on the basis of this standard. Since the samples were marked manually and these samples were likely to be marked mistakenly, this was a limitation that has been mentioned in many previous studies.

On the basis of the five features, three class-action models were built by training three ML classifiers, namely, RF, SVM, and BP. The SVM classifier was superior to the RF and BP classifiers, as shown in Table 6, since it provided better performance in terms of AUC, error rate, sensitivity, and specificity. These results were shown to be convincing through fivefold confirmation, which was consistent with the findings of previous studies (Zhang et al., 2017). The model in this article was superior to the previous models since it depended on only five features while showing the same AUC. Since the software that is needed to perform texture analysis and build classification models is readily available, clinicians can easily perform such analyses in clinical settings.

This study had some limitations. First, the dataset was modified, since the model was trained with only 80 samples. Its robustness needs further examination. Second, some degree of selection bias may exist. Different categories of brain tumors have different texture features. Some unique features were excluded, which may have influenced the results of our analysis. Third, a manual approach was adopted to segment tumors in this study. Although manual segmentation generally works better than automatic methods, segmentation errors could still exist. Some noise dots may have been mistakenly marked manually, negatively influencing the formation of our model.

## Conclusion

In conclusion, we hypothesized that a few of the textures acquired from the MRI images could serve the role of classification features, thereby significantly improving the classification efficiency. The Gini impurity index in the RF was applied to select features. On the basis of the five features, three class-action models were built by training three ML classifiers, including RF, SVM, and BP. The classifier model in this article was superior to the previous models, since it depended on only five features. On the basis of our initial findings, tumor regions characterized on the basis of MRI-based texture analysis may have clinical usefulness in differentiating brain tumors.

## Data Availability Statement

The datasets presented in this article are not readily available because the data cannot be used out of the hospital. Requests to access the datasets should be directed to med.info@njmu.edu.cn.

## Ethics Statement

The studies involving human participants were reviewed and approved by the Medical Ethics Committee of Nanjing Medical University. The patients/participants provided their written informed consent to participate in this study.

## Author Contributions

All authors listed have made a substantial, direct and intellectual contribution to the work, and approved it for publication.

## Conflict of Interest

The authors declare that the research was conducted in the absence of any commercial or financial relationships that could be construed as a potential conflict of interest.
